# A case of severe liver injury due to hepatitis B virus-related immune reconstitution inflammatory syndrome following HIV treatment reinitiation: diagnosis by liver biopsy

**DOI:** 10.1186/s12981-025-00838-1

**Published:** 2026-01-30

**Authors:** Koko Shibutani, Nobuyoshi Mori

**Affiliations:** Division of Infectious diseases, St. Luke’s International Hospital, 9 − 1 Akashi-Chuo, Chuo-ku, Tokyo, 104–8560 Japan

**Keywords:** HBV, HIV, IRIS, HBV-flare

## Abstract

**Background:**

HIV and HBV frequently coexist, with an estimated 8% prevalence of chronic HBV among people living with HIV (PLWH). In profoundly immunosuppressed PLWH, initiation or reinitiation of antiretroviral therapy (ART) can trigger immune reconstitution inflammatory syndrome (IRIS). When directed against HBV, IRIS can manifest as a hepatic flare (IRIS-HF). The long-term clinical implications of IRIS-HF remain incompletely understood.

**Case presentation:**

We describe a 42-year-old man with HIV/HBV coinfection who had discontinued ART for one year. On ART reinitiation with bictegravir/emtricitabine/tenofovir alafenamide, his CD4 count was 2.3 cells/µL and HIV RNA was 4.8 × 10⁵ copies/mL. Five weeks later, he developed a severe hepatic flare (AST 987 U/L, ALT 968 U/L). The differential diagnosis included HBV-related IRIS, opportunistic infections (CMV hepatitis, disseminated MAC, EBV-associated lymphoma), and drug-induced liver injury. A liver biopsy revealed fatty degeneration and lymphocytic infiltration, consistent with HBV-related IRIS. Transaminases normalized by week 11. He subsequently achieved HBsAg loss with anti-HBs seroconversion within 2 years after ART reinitiation.

**Conclusion:**

This case illustrates HBV flare due to IRIS following ART reinitiation in a profoundly immunosuppressed patient. The subsequent HBsAg loss suggests that IRIS-HF may promote HBV clearance, highlighting its potential role in achieving a functional cure. Vigilant monitoring of liver function is essential during ART initiation or reinitiation in HIV/HBV coinfected individuals.

## Background

HIV (Human Immunodeficiency Virus) and HBV (Hepatitis B Virus) coinfection remains an important clinical challenge due to overlapping transmission routes and compounded morbidity. In Japan, where most people living with HIV (PLWH) are men who have sex with men (MSM), HBV coinfection or prior exposure is frequent, with an estimated chronic HBV prevalence of up to 8% among PLWH [1, 2].

Immune reconstitution inflammatory syndrome (IRIS) can complicate antiretroviral therapy (ART) initiation or reinitiation, particularly in patients with advanced immunosuppression. In HIV/HBV coinfected patients, IRIS can trigger an HBV-related hepatic flare (IRIS-HF), characterized by abrupt elevations in aminotransferases. However, opportunistic infections and drug-induced liver injury can mimic this presentation, complicating diagnosis.

The long-term impact of IRIS-HF on HBV remains poorly defined; therefore, we present a case in which liver biopsy was crucial for diagnosis.

## Case presentation

The patient was a 42-year-old MSM with HIV/HBV co-infection previously, who had been treated with bictegravir/emtricitabine/tenofovir alafenamide (BIC/FTC/TAF). He had discontinued ART for one year without sexual exposure during that period.

He presented with seizures. CT (computed tomography) brain without contrast and contrast-enhanced MRI (magnetic resonance imaging) and electroencephalography (EEG) showed no epileptic lesions. Cerebrospinal fluid (CSF) analysis revealed an opening pressure of 17 cmH₂O, with a clear and colorless appearance. The cell count was ≤ 1, protein concentration was 42 mg/dL, and glucose level was 62 mg/dL. The blood glucose level at the time of lumbar puncture was 108 mg/dL. CSF studies, including bacterial culture and mycobacterial cultures, Film Array ME panel, cryptococcal-Ag, adenosine deaminase (ADA), and PCR for BK virus, JC virus, cytomegalovirus (CMV), and Epstein–Barr virus (EBV), were all negative.

At ART reinitiation, his CD4 cell count was 2.3 cells/µL and his HIV-RNA was 4.8 × 10^5^ copies/mL. He was promptly restarted on BIC/FTC/TAF, and prophylaxis for pneumocystis pneumonia with trimethoprim and sulfamethoxazole (TMP–SMX) was initiated. He had no ocular symptoms such as blurry vision or photophobia. Liver transaminases had been within the normal range before ART interruption but increased to AST 95 IU/L and ALT 73 IU/L. HBV serology showed HBsAg > 150,000 mIU/mL, HBeAg positivity, anti-HBeAb negativity, and HBV-DNA 8.4 log IU/mL.

Febuxostat was initiated for hyperuricemia but discontinued when liver enzymes worsened, and TMP–SMX was switched to atovaquone. Despite these changes, AST/ALT further increased to 987/968 IU/L. The longitudinal changes in liver enzymes and HBV serological markers are summarized in Table 1. At this point, Hepatitis C Virus (HCV) antibody (Ab) was negative, and HCV-RNA was not detected; hepatitis A IgM-Ab was also negative.

**Table 1:** The longitudinal changes in liver enzymes and HBV serological markers

Laboratory findings	Baseline	ART resumption	5 weeks after ART resumption	6 weeks after ART resumption	7 weeks after ART resumption	11 weeks after ART resumption	15 months after ART resumption	2 years after ART resumption
AST U/L	26	95	231	987	381	26		38
ALT U/L	31	73	213	968	651	24		22
Total bilirubin mg/dL	0.9	1.0	0.5	1.8	1.1	0.8		1.0
ALP U/L	156	124	227	268	270	114		105
HIV-RNA copy/mL	Not detected	4.8 × 10*5	2.9 × 10*2	1.1 × 10*2		5.6 × 10*1		Not detected
CD4 cell count µ/L	628.6	2.3	17.9	89.7		44.6		142.0
HBsAg	+	+					+	-
HBsAb	-						-	+
HBV-DNA LogIU/mL	Not detected	8.4	5.9	4.9		2.1	1.1	Not detected
HBeAg		+					-	-
HBeAb	-	-					-	-

**Fig. 1:**
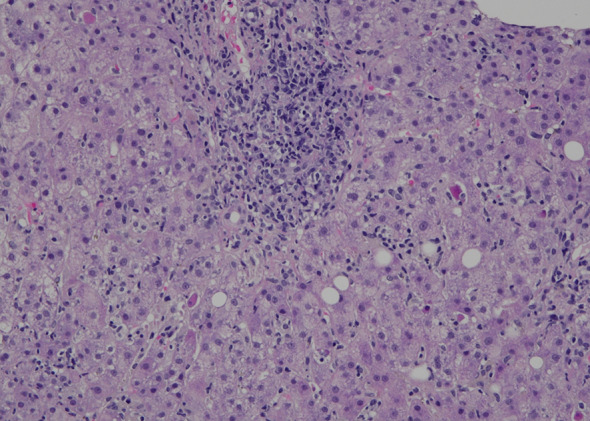
H&E stain of the liver biopsy specimen. The biopsy shows lymphocytic infiltration in the periportal area and around the central vein, fatty degeneration of hepatocytes, and obscuration of the limiting plate, findings consistent with immune-mediated inflammatory changes

The differential diagnosis included HBV flare, drug-induced hepatitis, CMV hepatitis, EBV-associated lymphoma, and disseminated Mycobacterium avium complex (MAC) infection. Bone marrow studies were negative for MAC, EBV, and CMV. Given that the CMV antigenemia assay detected 1 positive cell per 50,000 leukocytes, and the EBV DNA load was 3.26 log₁₀ copies/mL, a liver biopsy was performed to evaluate possible CMV hepatitis or EBV-associated lymphoma. Hematoxylin and eosin (H&E) staining revealed fatty degeneration and lymphocytic infiltration in the periportal area and around the central vein, with obscuration of the limiting plate in the portal region. Emperipolesis and pigment-phagocytic cells were also observed (Fig. 1). These findings were consistent with immune-mediated inflammatory changes rather than malignant or opportunistic infectious processes. Additional histological and immunohistochemical evaluations, including Azan staining, Ziehl–Neelsen staining, CMV immunostaining, and EBV-encoded RNA in situ hybridization, showed no evidence of fibrosis progression, mycobacterial infection, CMV infection, EBV infection, or malignancy. Transaminases normalized by week 11 (Table 1). Based on the liver biopsy findings and clinical course—characterized by worsening hepatic injury after ART initiation, followed by improvement—the episode was interpreted as an HBV flare due to IRIS after ART resumption following treatment interruption. At the time of liver enzyme normalization (fifteen months after re-initiation of ART), the HBV panel showed HBsAg positivity (quantitative value: 34 mIU/mL), negative HBeAg, negative anti-HBeAb, and an HBV DNA level of 1.1 log IU/mL. Seventeen months after treatment initiation, HBsAg became undetectable, and by 22 months, anti-HBsAb was detected, confirming successful seroconversion (Table 1).

## Discussion

This case describes a patient with profound immunosuppression after a one-year ART interruption who subsequently developed a hepatitis B flare consistent with IRIS upon ART resumption. IRIS is a recognized complication of ART initiation, particularly in patients with profound immunosuppression [3, 4]. In patients with HIV/HBV co-infection, HBV flares due to IRIS have been reported [5–7], and in untreated HIV/HBV co-infected patients, early HBV flares occur in approximately 22% of cases, typically within 56 days of ART initiation [8]. Although most reports describe HBV flares after initial ART initiation in untreated patients, our case is notable for its occurrence after the treatment reinitiation with a markedly reduced CD4 count and high HIV viral load, conditions that substantially increased the risk of IRIS. In addition, elevated ALT and high HBV-DNA at the time of ART initiation have been identified as predictors of IRIS-related HBV flares [8, 9]. In this case, ALT was 73 U/L and HBV DNA was 8.4 log IU/mL at resumption. Previous studies have suggested that higher baseline HBV DNA levels are associated with hepatic flares after antiretroviral therapy initiation, although no definitive cutoff has been established. In the TICO trial, patients with early hepatic flares had higher baseline HBV DNA levels than those without flares [8]. In this context, the HBV DNA level of 8.4 log IU/mL in our patient was considered relatively high based on comparison with previously reported data. Taken together, these findings indicate that in HIV/HBV co-infected patients, HBV flares must be carefully considered not only at initial treatment initiation but also at treatment re-initiation after interruption, particularly in the presence of low CD4 counts, high HIV-RNA levels, and elevated ALT or HBV-DNA levels.

The diagnosis in this case was confirmed by liver biopsy. Although HBV flare is relatively common after ART initiation in HIV/HBV co-infected patients, the differential diagnosis in profoundly immunosuppressed patients must include opportunistic conditions and drug-induced liver injury, which can present with similar hepatocellular damage. While fulminant hepatitis due to IRIS-related HBV flares in HIV/HBV co-infection is uncommon, the risk of coagulopathy would contraindicate liver biopsy in such cases. Therefore, when clinically feasible, early liver biopsy should be considered in patients with advanced immunosuppression and unexplained liver injury, both to establish the diagnosis and to exclude competing etiologies. Importantly, there are relatively few reports describing the histopathological features of HBV-related IRIS in HIV/HBV co-infection [5–7], and the performance of liver biopsy in this case provides valuable information that contributes to the accumulation of knowledge through future case reports. Nevertheless, this case has limitations, including the lack of tissue-based PCR assays using the liver biopsy specimen, which were not available at our institution and may have allowed a more definitive exclusion of alternative infectious etiologies.

Beyond diagnosis, this case adds to the growing evidence that HBV flares triggered by IRIS may favorably after the natural history of chronic HBV infection. A recent study demonstrated that HBsAg loss occurred far more frequently in patients who developed IRIS-HF (73%) compared with those who did not (21%), and IRIS-HF was identified as an independent predictor of subsequent HBsAg loss [10]. Furthermore, this study demonstrated that in the IRIS-HF group, 57% (8/14) achieved HBsAg loss within 3 years of ART initiation compared with only 15% (6/41) in the non-HF group, and that a rapid HBsAg decline of > 2 log IU/mL within 12 months was observed in the IRIS-HF group, whereas the decline was < 1 log IU/mL in the non-HF group [10]. Other case reports have also documented that hyperactivation of the immune system facilitated the control of HBV replication and contributed to HBsAg seroconversion during TAF/FTC therapy [11]. In line with previous findings, our patient with HIV/HBV co-infection developed an HBV flare due to IRIS and subsequently achieved HBsAg loss 22 months after ART reinitiation. Taken together, these observations further support the potential role of immunomodulatory agents in strategies aimed at achieving a functional cure of HBV.

## Conclusion

This case highlights an HBV flare due to IRIS following ART reinitiation in a profoundly immunosuppressed HIV/HBV-coinfected patient. Liver biopsy was pivotal in establishing the diagnosis, emphasizing its value in differentiating HBV flare from opportunistic infections or drug-induced liver injury when clinically feasible. The clinical course—characterized by hepatic flare followed by HBsAg seroconversion—supports previous observations that IRIS-related hepatic flare may facilitate HBV clearance and ultimately, contribute to functional cure.

## Data Availability

No datasets were generated or analysed during the current study.

## Abbreviations

PLWH: Ueople living with HIV
ART: Antiretroviral therapy
IRIS: Immune reconstitution inflammatory syndrome
IRIS-HF: Immune reconstitution inflammatory syndrome hepatic flare
Ag: Antigen
HIV: Human Immunodeficiency Virus
HBV: Hepatitis B Virus
MSM: Men who have sex with men
BIC/FTC/TAF: Bictegravir/emtricitabine/tenofovir alafenamide
CT: Computed tomography
MRI: Magnetic resonance imaging
EEG: Electroencephalography
CSF: Cerebrospinal fluid
ADA: Adenosine deaminase
CMV: Cytomegalovirus
EBV: Epstein–Barr virus
TMP–SMX: Trimethoprim and sulfamethoxazole
HCV: Hepatitis C Virus
Ab: Antibody
MAC: Mycobacterium avium complex
H&E: Hematoxylin and eosin
ISH: In situ hybridization
